# Inhibition of Tissue Factor Expression in Brain Microvascular Endothelial Cells by Nanoparticles Loading NF-κB Decoy Oligonucleotides

**DOI:** 10.3390/ijms9091851

**Published:** 2008-09-18

**Authors:** Huafang Wang, Yu Hu, Tao Guo, Heng Mei, Xiaoping Zhang, Wangqiang Sun

**Affiliations:** 1 Institute of Hematology, Union Hospital, Tongji Medical College, Huazhong University of Science & Technology, 1277 Jie-fang Dadao, Wuhan, Hubei 430022, P.R. China; 2 Laboratory of Targeted Biotherapy, Huazhong University of Science & Technology, 1277 Jie-fang Dadao, Wuhan, Hubei 430022, P.R.China; 3 College of Chemical and Environmental Engineering, Hubei University of Technology, 634 Xiongchu Avenue, Wuhan, Hubei 430068, P.R.China

**Keywords:** Tissue factor, decoy oligonucleotides, nanoparticles, nuclear factor-kappaB, polylactic acid

## Abstract

To investigate a nuclear factor-kappa B decoy oligonucleotides strategy on the inhibition of tissue factor (TF) expression in cultured rat brain microvascular endothelial cells (BMECs) by polylactic acid (PLA) nanoparticles delivery system and to evaluate this new vector for *in vitro* gene therapy. Nanoparticles were formulated using poly D,L-polylactic acid with surface modifying by polysorbates 80. 3-[4,5-Dimethylthiazol-2,5-diphenyl-2*H*-tetrazolium bromide] (MTT) assays showed that PLA nanoparticles were not toxic to the cultured BMECs.The decoy oligonuceotides (ODNs) loaded within nanoparticles was 6 μg/mg, encapsulation efficacy was (60.5±1.5)%. It was observed by flow cytometry that the cellular uptake of nanoparticles depended on the time of incubation and the concentration of nanoparticles in the medium. And confocal microscopy demonstrated that nanoparticles localized mostly in the BMECs cytoplasm. The released decoy oligonuceotides (ODNs) uptaked by BMECs retained their biologic activity and led to reduced level of tissue factor expression as compared to control cultures. These findings offer a potential therapeutic strategy in the control of TF expression in BMECs *in vitro* and suggest that PLA nanoparticles may be appropriate as delivery vehicles for decoy strategy in the gene therapy of cerebral thrombosis.

## 1. Introduction

Thrombosis plays a major role in the pathogenesis of ischemic stroke. Although some limited progress has been made in the treatment of cerebral thrombosis, current therapeutic options are still controversial because of a discouragingly high incidence of intracerebral hemorrhage and restenosis in treated patients [1–4]. Consequently, innovations for therapy of cerebral thrombosis have focused on selective and non-bleeding methods.

Abnormal tissue factor (TF) expression on vascular endothelial cells may account for thrombotic events associated with cardiovascular and cerebrovascular disease [5–7]. *In vitro*, a variety of inflammatory stimuli and immunological mediators, including bacterial endotoxin (lipopolysaccharide, LPS), the tumor necrosis factor-α (TNF-α) and interleukin-1β (IL-1β), can change endothelial cells to a procoagulant state by inducing TF expression.

TF expression in vascular endothelial cells exposed to LPS can be regulated at level of transcription [8]. The activation of transcription factor nuclear factor-kappa B (NF-κB) plays a key role in TF expression in damaged endothelial cells [9]. In recent studies, we have identified NF-κB transcription factor decoy (TFD) strategy, which is believed to be a powerful tool for antigene strategies and transcription regulation [10–12], and can inhibit TF over-expression in stimulated human umbilical vein endothelial cells (HUVEC) by disruption of NF-κB activation [13]. Therefore, the decoy approach may enable us to prevent cerebrovascular diseases once oligonuceotides (ODNs) was delivered into brain.

Brain microvascular endothelial cells (BMECs) were important targets in gene therapies for cerebral thrombosis. We have reported that polysorbate 80 (Tween 80, T-80)-coated nanoparticles (NPs) can be used as drug delivery vehicle targeting brain vessels [14–15]. The mechanism suggests that drug-loaded NPs may be endocytosised by BMECs. Thus, the presence of this mechanism may provide an efficient way in the brain vascular endothelial cells targeting.

In this study, we examined the ability of NF-κB decoy ODNs enveloped in polylactic acid (PLA) NPs to suppress TF gene expression in primary cultures of rat BMECs. The data offered a potential therapeutic strategy in the regulation of TF expression in BMECs *in vitro* and suggest that PLA NPs may be an appropriate delivery vehicle for decoy strategy in the gene therapy of cerebral thrombosis.

## 2. Materials and methods

### 2.1. Materials

PLA (racemic, 5000 in molecular weight) was purchased from Institute of Medical Equipments of Shandong Province, P.R. China. Reagents were analysis-grade chemicals made in China, mainly including polyvinyl alcohol (PVA), cetyltrimethyl ammonium bromide (CTAB), dimethyl sulfoxide (DMSO) and paraformaldehyde. Endothelial cell growth supplement (ECGS), collagenase II, MTT and LPS were purchased from Sigma Chemical Company. Culture media Dulbecco’s Modified Eagle Media (DMEM), fetal bovine serum, penicillin-streptomycin, and Trizol Reagent were obtained from GIBCO-BRL, Life Technologies. Anti-p65 rabbit polyclonal antibody was obtained from Santa Cruz Biotechnology. Taq DNA polymerase and Moloney Murine Leukemia Virus (MMLV) reverse transcriptase 1-kb DNA ladder were from Promega Corporation. Neonate Wistar rats were supplied by the Experimental Animal Center of Tongji Medical College, Huazhong University of Science & Technology, Hubei, P.R. China.

### 2.2. Culture of BMECs

The method of primary culture of rat BMECs has been previously described [16,17]. In brief, cerebral microvessels were collected from neogenetic Wistar rats by homogenate and twice filtrations. Microvessels were dissociated with 0.2% collagenase II by steady shaking for 10min at room temperature. The endothelial cells were collected after differential centrifugation and filtering through nylon mesh, resuspended in the DMEM medium, and supplemented with 10 mmol/L Hepes, 20% fetal calf serum, 20 mg/L ECGS, 100 mg/L heparin, 100 unit/mL penicillin, and 100 μg/mL streptomycin. After filtrated through nylon mesh, BMECs were plated onto collagen coated Petri dishes for 4h, allowing for BMECs attachment to the collagen substrate. And then the media was removed. Afterward, the media was changed twice a week.

### 2.3. Decoy design

Upper-strand and reverse-complement phosphorothioated oligonucleotides were commercially synthesized, purified and annealed by Saibaisheng Inc. (Shanghai, China). ODNs used for the study were phosphorothioate-modified to reduce intracellular nuclease digestion. For fluorescence studies, ODNs were 5′-end-labeled with fluorescein isothiocyanate (FITC). Two ODNs sequences were synthesized and used. The NF-kappa;B decoy ODNs has the same sequence with the κB site in TF promoter, which is 5′-GTCCCGGAGTTTCCTACCGGG-3′ with the consensus sequence underlined. In addition to the NF-κB decoy ODNs, a scrambled decoy was used as control for specificity. The sequence of scrambled decoy (M-decoy) was different from the κB site in the consensus sequence while the flanking sequence was the same, which is 5′-GTCCATCCTGGGATTACCGGG-3′.

### 2.4. Formulation of PLA NPs containing NF-κB decoy ODNs

Firstly, decoy ODNs were modified with the cationic surfactant CTAB to improve the lipophilicity. ODNs loaded PLA NPs were then prepared by a nanoprecipitation method [14, 18] with some modifications. Briefly, ODNs-CTAB was dissolved in DMSO and then slowly added into PLA solution. The pre-formed suspension was slowly dripped into 2.5% (wt/V) poly (vinyl alcohol) (PVA) solution where the ratio of DMSO to PVA was 1:4–1:6.6(V/V). Centrifuged the suspension three times to remove the DMSO and PVA. The fabricated NPs were finally collected, centrifuged, lyophilized to dryness, and stored at −70 °C before use. The procedure was applied to both decoy and mutant ODNs.

The initial total encapsulation amount measuring of ODNs-nanoparticles: ODNs-PLA NPs powder (decoy PLA NPs and M-decoy-PLA NPs, 20 mg) was dissolved in methylene dichloride (1 mL), kept in 37 °C for 6 hours, PBS (1 mL) was added to the solution. Twnty four hours later, the ODNs was supposed to be resolved in the solution sufficiently and its concentration was measured by the fluorospectrophotometer. Experiments were performed in triplicate.

### 2.5. Particle size analysis and zeta potential

The morphology of PLA NPs loading ODNs was observed with atomic force microscopy (AFM) in the tapping mode. A drop of the suspension was dripped to the freshly cleaved mica and dried at room temperature. AFM observations were then done with a SPA 400 AFM (Seiko Instruments Industry Co., Ltd. Japan). The polydispersity index and the zeta potential of PLA NPs were measured in triplicate by a Zetasizer (Zeta Pals, Brookhaven Instruments, USA) at 15 °C at a wavelength of 635.0 nm.

### 2.6. Cellular toxicity of PLA nanoparticles:

MTT assay was employed to evaluate the cellular toxicity of prepared PLA nanoparticles. The cells in exponential growth phase were seeded in a 96-well plate making sure that 1×10^5^ cells were present in each well. PLA nanoparticles suspension was added to each well and the final concentration should be 1, 2, 5, 10, 20 μg/mL. The incubation duration for each concentration group should be 30 min, 1 h, 2 h, 4 h, 8 h, 16 h, respectively. Each group should include 3 to 5 wells. 6 wells were taken as normal cell group and another 6 wells were taken as blank group (only cell culture fluid included).The plate was incubated at 37 °C in a humidified incubator, 5% CO_2_ for 48 hours. Then MTT reagent (5 mg/mL, 20 μL) was added into each well and the plate was incubated at 37 °C for 4 hours. The the medium was removed and DMSO (200 μL) was added to each well. After 1 hour incubation, absorbance(A) at 490 nm was measured using a plate reader.The cell growth inhibitory ratio was calculated via following formula and described with cell growth inhibitory curve. The half cell growth inhibitory concentration (IC50) was calculated with the curve. Cell Growth inhibitory ratio=[1 - (average absorbance of experimental group/average absorbance of contrast group)]×100%.

### 2.7. Detection the uptake rate of PLA NPs loading ODNs in BMECs

Confluent BMECs were digested by 0.02% EDTA plated into 24-well multidishes at a density of 4×10^4^ cells per well. Cells were kept in medium DMEM + 10% Fetal Bovine Serum (FCS) for 24 h at 37 ºC, 5% CO_2_ before use. Columns accommodated the following formulations: (1) suspension of blank PLA NPs, (2) cell cultures exposed to 5 μmol/L ODNs in PBS solution, (3) cell cultures exposed to mixture of 5 μmol/L ODNs and PLA NPs, (4–6) cell cultures exposed to decoy ODNs loaded PLA NPs with ODNs concentration of 0.5, 2.5, and 5 μmol/L in medium. Row provided for 4 independent data points per iteration. One single array was harvested at each of 30 min, 60 min and 120 min time period. Cells were detached from culture plates with EDTA solution and washed twice with PBS. The percentage of FITC-positive cells was detected by flow cytometric analysis at a wavelength of 490 nm.

### 2.8. Observation of cellular localization of ODNs loaded PLA NPs

BMECs were plated into dishes in which have sterile slides and were maintained in medium as above described. The FAM labelled ODNs-PLA NPs were added for 1 h after replating. The control cells were incubated with NPs without ODNs. Then medium was removed and acetone was added to fix the cells. Slides were washed three times with PBS and observed under confocal microscope. The excitation wave and emission wave of optical filter were 490 nm and 520 nm respectively.

### 2.9. Expression of TF mRNA in BMECs induced by NF-κB decoy ODNs loaded PLA NPs

BMECs were plated in 24-well arrays of 4-row × 6-column. Columns accommodated the following formulations: (1) suspension of blank NPs, (2) cell cultures exposed to 5 μmol/L NF-κB decoy ODNs in PBS solution, (3) cell cultures exposed to M-decoy ODNs loaded PLA NPs with ODNs concentration of 0.5 μmol/L, (4) cell cultures exposed to NF-κB decoy ODNs loaded PLA NPs with ODNs concentration of 0.5 μmol/L, (5) cell cultures exposed to mixture of 0.5 μmol/L ODNs and PLA NPs, (6) control of blank. Row provided for 4 independent data points per iteration. ODNs were added 6 h after re-plating and maintained for 1 h before LPS (1 μg/mL) was added in.

The mRNA expressions of TF and glyceraldehydes phosphate dehydrogenase (GAPDH) in BMECs were semiquantitatively detected by reverse transcription-polymerase chain reaction (RT-PCR). BMECs were collected after exposed to LPS for 2 h. Total RNA was then isolated from untreated control cells, cells stimulated with LPS, and decoy-transferred cells, respectively, by utilization of the TRIZOL reagent following the manufacturer’s instructions (Gibco). 10 to 12 μg of total RNA could be yielded from 5×10^5^ cells. RNA samples were diluted to a final concentration of 1μg/μL in RNase-free water and stored at –80 ºC until use. Synthesis of cDNA was performed with 1 μg of total RNA. The 20×RT reaction consisted of 5× firststrand buffer, 0.5 mmol/L dNTP, 50 nmol/L random primers, and 20 U Superscript reverse transcriptase (all reagents from Promega, Madison, Wisconsin, USA). PCR reaction was carried out with specific primers. For TF, the sense primer was 5′-CTA CTG TTT CAG TGT TCA AGC AGT GA-3′ and the antisense primer was 5′-CAG TGC AAT ATA GCA TTT GCA GTA GC-3′, with an amplified product of 283bp. For GAPDH, the sense primer was 5′-CAA AGT TGT CAT GGA TGA CC-3′ and the antisense primer was 5′-CAA TGG AGA AGG CTG GGG-3′, with an amplified product of 195bp. PCR reaction was performed with 30 cycles, with denaturation at 94 °C for 45 s, annealing at 58 °C for 45 s and extension at 72 °C for 45 s. At the end of these cycles, a further extension was carried out at 72 °C for 10 min. Negative controls as no cDNA or no RTase in the reaction were used to make sure that there was no artificial amplification and to exclude amplification of genomic DNA. The PCR products were separated on a 1% agarose gel containing 0.01% ethidium bromide and the intensities of the stained bands were quantitated by densitometric analysis. DNA ladder was employed as the DNA marker. The amount of TF mRNA was normalized relative to the GAPDH mRNA.

### 2.10. Nuclear protein P65 in BMECs induced by decoy ODNs NPs

Western blot analyses were performed in order to investigate the level of P65 in nuclear of BMECs. BMECs were grouping and disposed as described in method 2.9. Nuclear protein was extracted according to the procedure of the kit (Pierce Biotechnology) after BMECs were exposed to LPS for 5h. Protein concentration was measured by bicinchoninic acid (BCA) method. Thereafter, 15μL extracted proteins were diluted in Laemmli sample buffer, denatured at 90 °C for 5 min, separated on 12% SDS-PAGE gels at 150 V for 45 min, and transferred to polyvinylidene difluoride (PVDF) membranes. Following transfer, PVDF membranes were incubated in blocking solution [Tris-buffered saline containing 5% (w/v) skim milk powder and 0.5% (v/v) Tween 20] for 2h at room temperature. Membranes were incubated with primary antibodies diluted in blocking solution overnight at 4 °C. Following primary incubation, membranes were washed three times in blocking solution and incubated for 1h with horseradish peroxidase-conjugated anti-IgG (Bio-Rad). A final wash over 30min with six changes in wash buffer (Tris-buffered saline containing 0.5% Tween 20) was performed. Immunoreactive bands were finally visualized by autoradiography using enhanced chemiluminescence (ECL) with exposure time of 30s to 2min. Densitometry was performed using Kodak EDAS 1D image analysis software.

### 2.11. Statistic analysis

The results were given as mean ± standard deviation. Data were stored and analyzed by the software package SPSS 11.5. Differences in means of normally distributed data were assessed by Student’s t test with Bonferroni correction. A *P* value less than 0.05 is considered significantly.

## 3. Results

### 3.1. Physical Characteristic of decoy ODNs loaded PLA NPs

**Figure 1.**
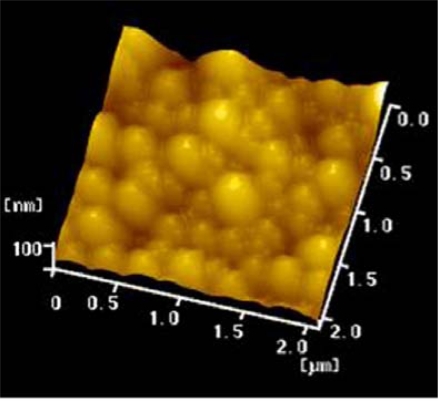
AFM image of PLA-NPs loading decoy ODNs.

**Figure 2.**
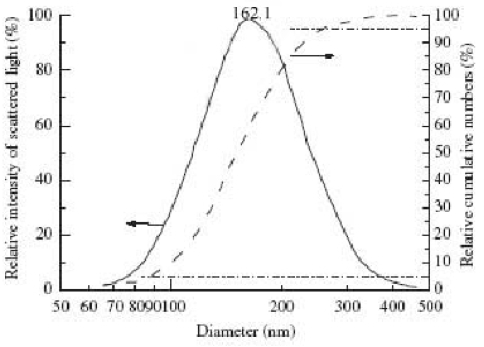
Size distribution of SFNPs: — density distribution and cumulative distribution.

The PLA NPs produced were uniform spherical particles with an effective diameter of 162.1 nm and a polydispersity index of 0.118 (Figure 1, Figure 2). The average surface zeta potentials of decoy ODNs NPs was 0.21 mV. Compared with blank PLA-NPs, which had a average zeta potentials of −17.37 mV, the zeta potentials of decoy ODNs NPs increased by 17.58 mV, suggesting that decoy ODNs were bound to PLA NPs, and the surface hydrophilicity of PLA NPs was obviously enhanced.

The initial total encapsulation amount of decoy-ODNs in NPs is 6 μg/mg,which is measured by spectrophotofluorometer.The corresponding encapsulation ratio is (60.5±1.5)% equivalently encapsulation.

### 3.2. MTT result

As Table 1 showed.The growth inhibitory ratios of BMECs by PLA NPs were very low, the highest was 8.4±1.8% with the corresponding concentration 20 μg/mL. There is a rising tendency of the BMECs growth inhibitory ratios when the working concentration increased. Analysis of variance showed in the same working duration, 1, 2, 5, 10 μg/mL concentration groups differed from each other significantly. The above results showed cytotoxicity of PLA on BMECs was very low. The cell growth inhibitory ratio didn’t increase when the working duration prolonged.These initial results showed rather high bioseurity of PLA.

### 3.3. PLA NPs based strategies for decoy ODNs delivery into BMECs

**Figure 3.**
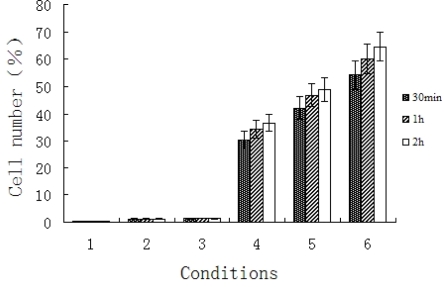
The efficiency of uptake of nanoparticles by BMECs in six varying groups. (1) blank PLA nanoparticles; (2) FITC labeled decoy ODNs in PBS; (3) mixture of decoy ODNs and PLA nanoparticles; (4) decoy ODNs-loaded nanoparticls in three varying concentrations (0.5, 2.5, and 5μM; in culture medium).

**Figure 4.**
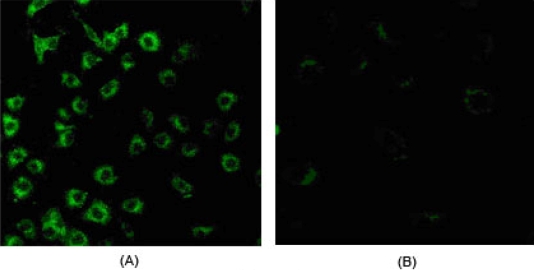
(A) Location of decoy-PLA nanoparticlesuptaked by BMECs in cytoplasma; (B) control cells were incubated with NPs without ODNs.

Decoy ODNs could be introduced efficiently into BMECs with the encapsulation of PLA NPs. NF-κB decoy ODNs encapsulated by PLA NPs (Group 4, 5, 6) found in BMECs by flow cytometry increased significantly compared to other control groups (Group 1, 2, 3 ; P<0.01, Figure 3). It was observed that there was bright green fluorescence in cytoplasma of BMECs exposed to decoy ODNs loaded PLA NPs (Figure 4A), while no fluorescence was seen in control cells exposed to PLA NPs without ODNs (Figure 4B).

### 3.4. Effect of NF-κB decoy ODNs loaded PLA NPs on the expression of TF mRNA in BMECs

**Figure 5.**
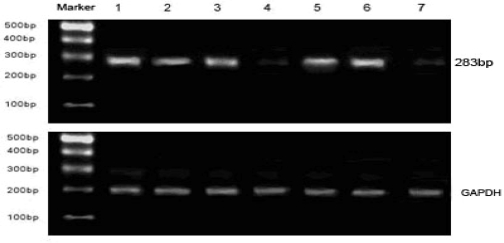
Expression of TF mRNA in BMECs stimulated by LPS exposed to different decoy-PLA nanoparticles. (1) blank PLA nanoparticles were added; (2) 0.5 μM; decoy ODNs in PBS were added; (3) PLA nanoparticles loading 0.5μM M-decoy were added; (4) PLA nanoparticles loading 0.5 μM NF-κB decoy ODNs were added; (5) mixture of decoy ODNs and PLA nanoparticles were added; (6) blank control:no nanoparticles were added; (7) BMECs without LPS stimulation.

Two hours after 1μg/mL LPS stimulation, TF mRNA levels were clearly increased (Lane 6 in Figure 5) than BMECs without stimulation (Lane 7 in Figure 5). In BMECs exposed to NF-κB decoy ODNs loaded PLA NPs, a significant reduction in TF mRNA was observed (Lane 4). In contrast, disposal with mutant decoy ODNs or PLA NPs alone had no effect on the expression of TF mRNA (Lane 1, 2, 3, 5) compared to TF mRNA level in BMECs exposed to LPS (Lane 6).

### 3.5. Effect of NF-κB TFD ODNs loaded PLA NPs on the level of P65 in nuclear of BMECs

**Figure 6.**
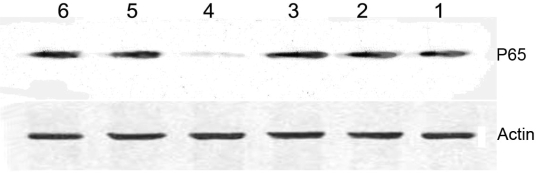
The change of content of P65 in BMECs nuclear extracts by preincubation with decoy-PLA nanoparticles. (1) blank PLA nanoparticles; (2) 0.5μM decoy ODNs; (3) M-decoy loaded PLA nanoparticles with 0.5μM M-decoy; (4) decoy-PLA nanoparticles with 0.5 μM NF-κB decoy ODNs; (5) mixture of decoy ODNs and PLA nanoparticles; (6) blank control.

Western blot analysis showed that P65 level in nuclear of BMECs, which were treated with LPS in the presence of decoy loaded PLA NPs, was significant lower than other groups (Figure 6).

## 4. Discussions

The strategy of cis decoy ODNs could regulate endogenous transcription of genes by binding or trapping transcription factors to deprive their functions. It becomes a new therapeutic method and a useful tool in the study of transcription regulation. In previous study, E2F decoy has been employed successfully in preventing intima hyperplasy of patients who had vein transplantation [19]. The decoy strategy in our study is to design a piece of oligonucleotide which has the same core sequence with the binding sites of cis element κB (5′CGGAGTTTCC 3′) in the promoter of TF. Binding of liberated NF-κB to phosphorothioated decoy deca-nucleotide elements within the cytoplasm prevents DNA binding and transactivation by the transcription factor of TF. It has been demonstrated that NF-κB decoy can obviously inhibit TF expression in endothelial cells stimulated by inflammatory factors and decrease the level of activated coagulation factor VII(FVIIa) by preventing activation of FVII indirectly. Thus, the hypercoagulable state (e.g. angina) may be retrieved and thrombogenesis may be diminished.

It is probable that such molecular therapeutic strategies will involve local delivery of NF-κB decoy ODNs. Even if a potential therapy is effective, it may not work because appropriate delivery has not been achieved. Though there are various vectors for transporting decoy ODNs, such as liposome and polycation delivery system, none of them can achieve for requisition of therapy of cerebrovascular diseases. Our work showed that the delivery system manufactured by polylactic acid nanoparticels could target cerebrovascular endothelial cells in animal experiments. For degradation products from PLA in bodies are carbon dioxide (CO_2_) and H_2_O [20], both with no harmful effects on organisms, PLA nanoparticle delivery system provides a method for gene therapy of cerebrovascular diseases in future.

Decoy ODNs must be condensed and encapsulated in nanoparticels before transported into cells. A major advantage of the modified nanoprecipitation method is the avoidance of violent stirring for long time to disperse the microparticles. Such a method can take more advantage in keeping the structure and activity of biomacromolecule than the method of emulsify-solvent volatilixation [21, 22]. To avoid strong mechanical action and poison solvent, decoy ODNs were modified with the cationic surfactant CTAB to improve the lipophile. Modified ODNs showed some positive charge, but the surface of ODNs loaded PLA NPs was nearly electroneutral and the zeta potentials were much higher than PLA NPs without ODNs loading. We also measured that the entrapment efficiency of ODNs was 60.5 ± 1.5. These findings indicate that a large amount of double-strand ODNs have incorporated to PLA NPs. Moreover, the PLA NPs we prepared were uniform spherical particles observed by AFM. These characteristics manifest that PLA NPs are appropriate as delivery vehicles for transporting ODNs into target cells.

BMECs are important target cells in the study of gene therapy for cerebrovasular diseases. It has been proved that NF-κB decoy ODNs could suppress TF expression in HUVECs in our previous study. In the present work, the level of TF mRNA in BMECs, uptaking NF-κB decoy ODNs loaded PLA NPs, was obviously lower than other control cultures. Furthermore, nuclear expression of NF-κB was found reducting significantly compared to control groups. These results provide the following evidence: (1) it is similar in cultured BMECs as other endothelial cells that TF is over expression when cells were stimulated by LPS; (2) NF-κB decoy ODNs released from those PLA NPs entering into BMECs were protected from degradation and remained biologically active and could inhibit TF expression.

## 5. Conclusions

Our results suggest that the molecular approach is effective *in vitro* to inhibit over-expression of TF in LPS stimulated BMECs and appropriate as delivery vehicles for NF-κB decoy ODNs. Further investigation on the application of PLA NPs loading ODNs for gene therapy *in vivo* are ongoing. The NF-κB decoy loaded nanoparticle will take an important role in the gene therapy and would provide a new method for treatments of cerebrovascular and other hypercoagulable diseases.

## Figures and Tables

**Table 1:** The cell growth inhibitory ratio of BMECs by PLA NPs (%)

working concentration (μg/ml)	Incubation time					
	30min	1h	2h	4h	8h	16h
1	1.5±0.8	1.8±1.7	2.5±1.2	1.8±1.0	1.6±1.2	1.6±1.1
2	1.8±1.1	1.7±1.2	2.0±1.4	2.1±0.9	1.9±1.6	1.8±2.1
5	3.6±1.5	2.7±1.5	2.5±1.2	2.8±1.2	2.0±1.9	1.9±1.6
10	5.2±1.6	4.0±1.3	3.6±0.2	3.2±0.3	2.8±1.1	2.2±1.2
20	8.4±1.8#	7.6±1.2#	6.7±2.2#	4.3±1.6#	3.8±2.1#	3.1±1.4#

# Comparison of cell growth inhibitory ratio between different working concentration in the same column of incubation time, p<0.05.
